# Supplementary material for: Promising Effect of a New Ketogenic Diet Regimen in Patients with Advanced Cancer
Source: Nutrients. 2020 May 19;12(5):1473. doi: 10.3390/nu12051473 (PMC7284721; doi:10.3390/nu12051473)
Supplement: Supplementary file 1 [file nutrients-12-01473-s001.pdf]

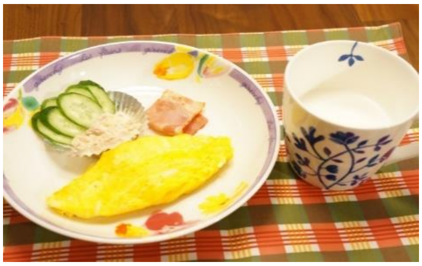

Breakfast
- Omelette (MCT oil)
- Tuna salad (MCT oil)
- Bacon
- ketogenic formula

Calorie	647 kcal
Protein	21.9 g
Lipid	60.9 g
Carbohydrate	3.9 g
Ketone ratio	2.3

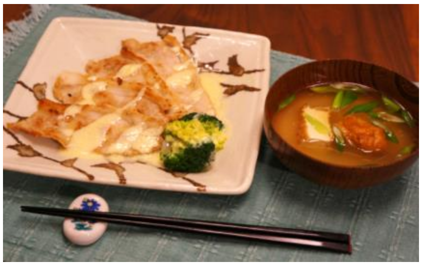

Lunch
- Saute (MCT oil)
- Miso soup (MCT oil)

Calorie	619 kcal
Protein	23.9 g
Lipid	56.1 g
Carbohydrate	3.4 g
Ketone ratio	2.1

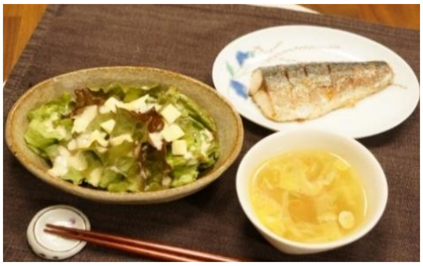

Dinner
- Grilled fish (mackerel)
- Consomme soup (MCT oil)
- Cheese salad (MCT oil)

Calorie	526 kcal
Protein	21.5 g
Lipid	48.5 g
Carbohydrate	2.7 g
Ketone ratio	2.0

Figure S1 : Examples of the ketogenic diet menu (carbohydrate 10 g/day).

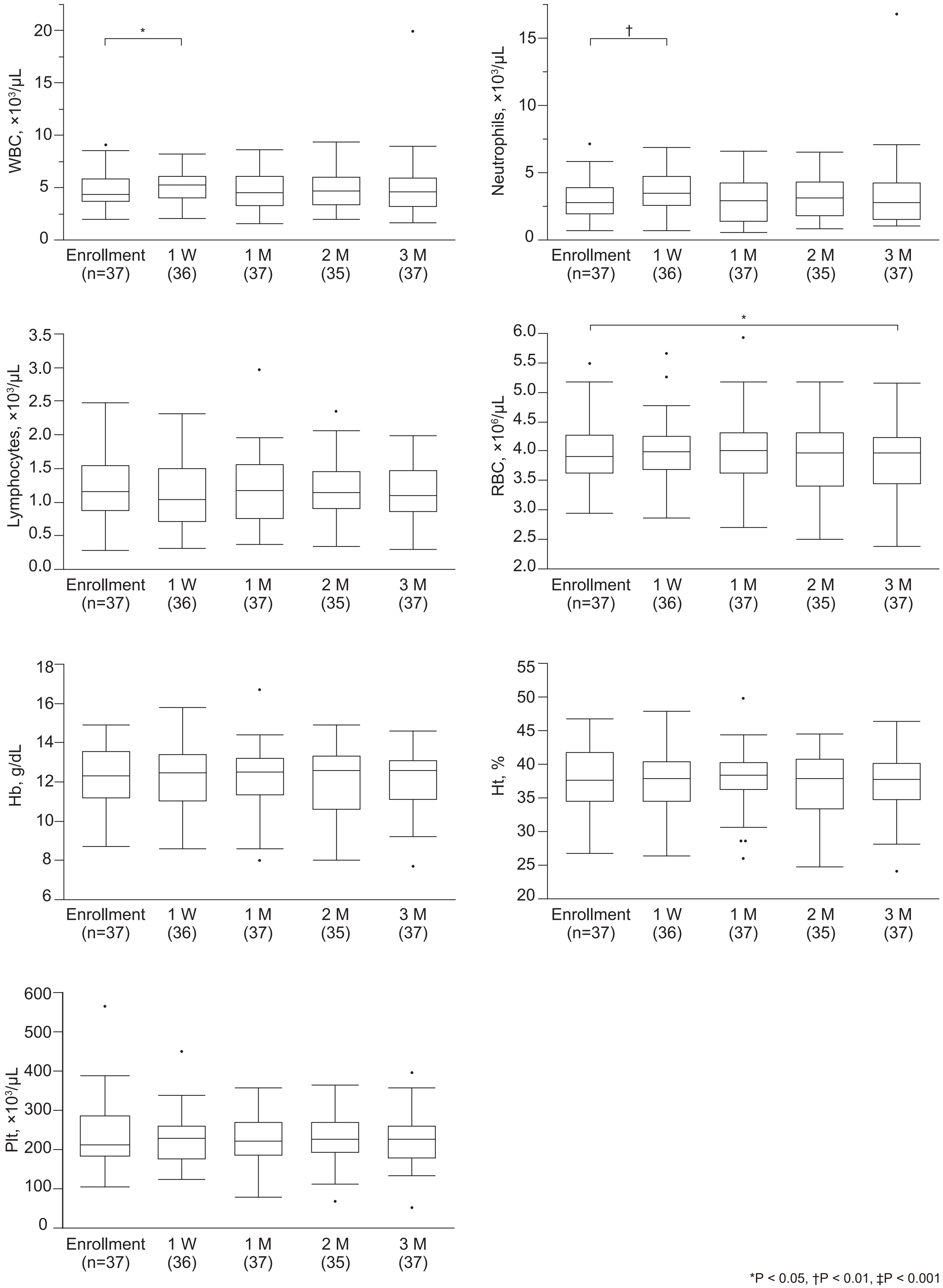

*P < 0.05, †P < 0.01, ‡P < 0.001

Figure S2-1 : Additional blood parameters of patients on the ketogenic diet.

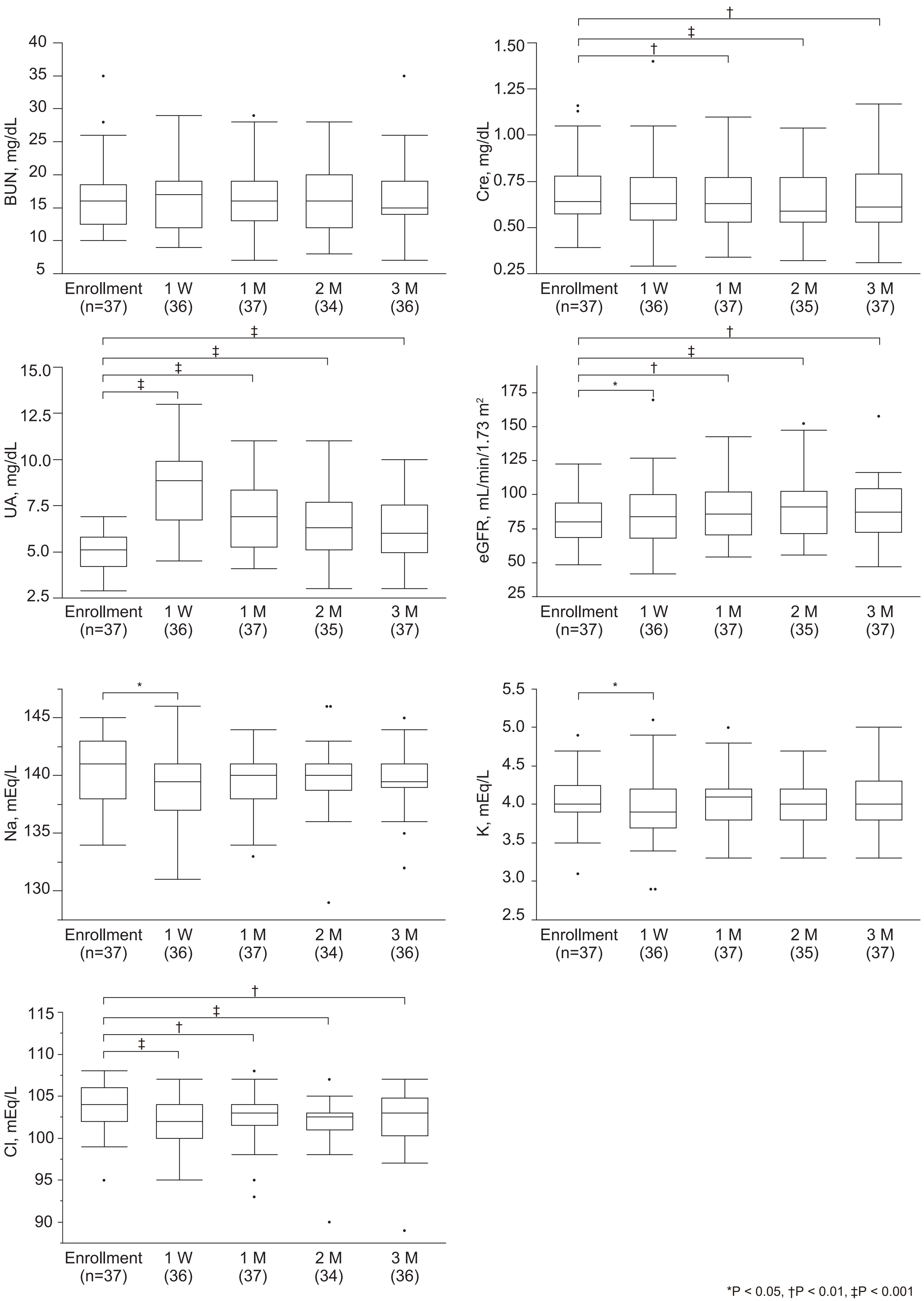

*P < 0.05, †P < 0.01, ‡P < 0.001

Figure S2-2 : Additional blood parameters of patients on the ketogenic diet.

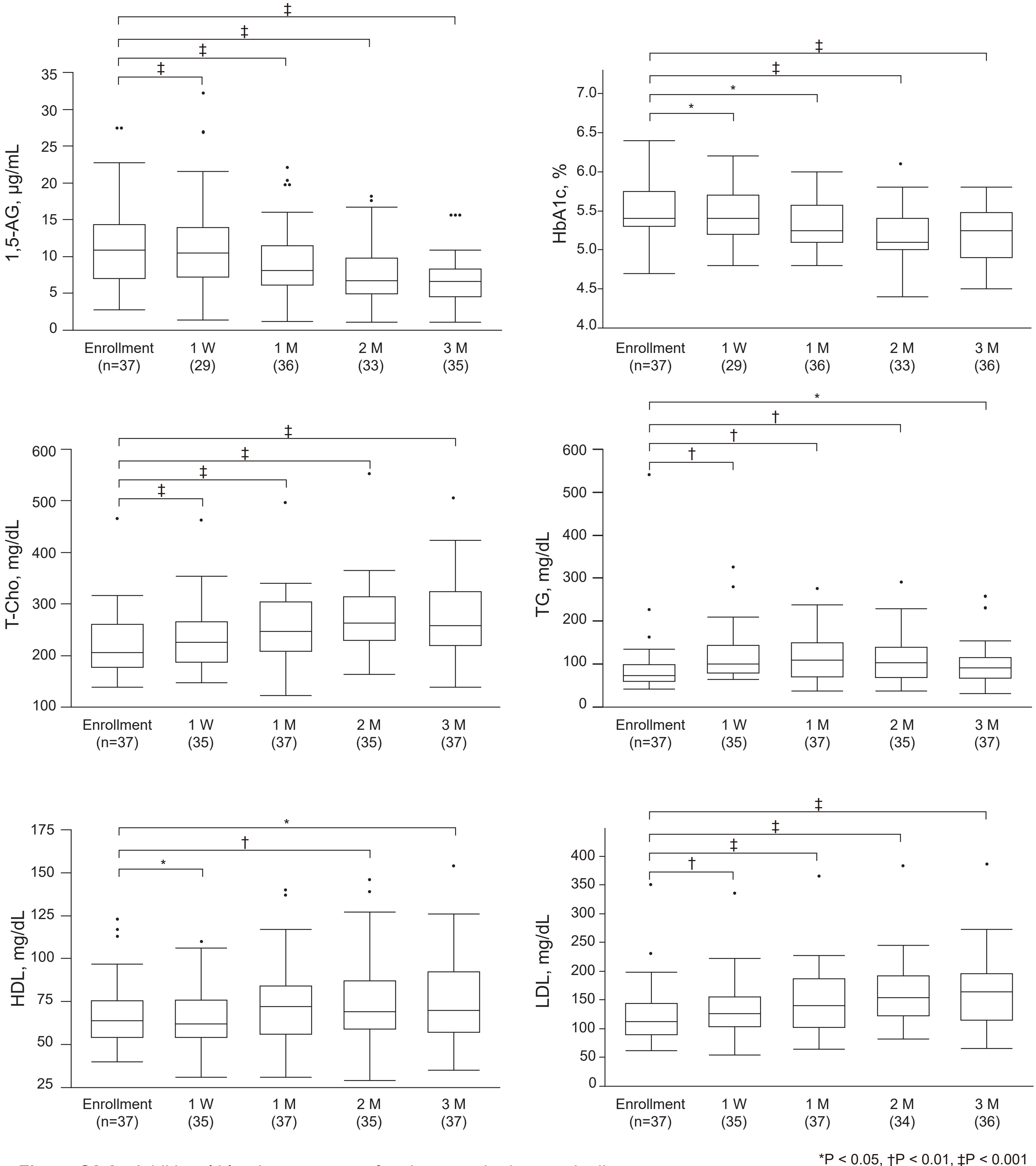

Figure S2-3 : Additional blood parameters of patients on the ketogenic diet.

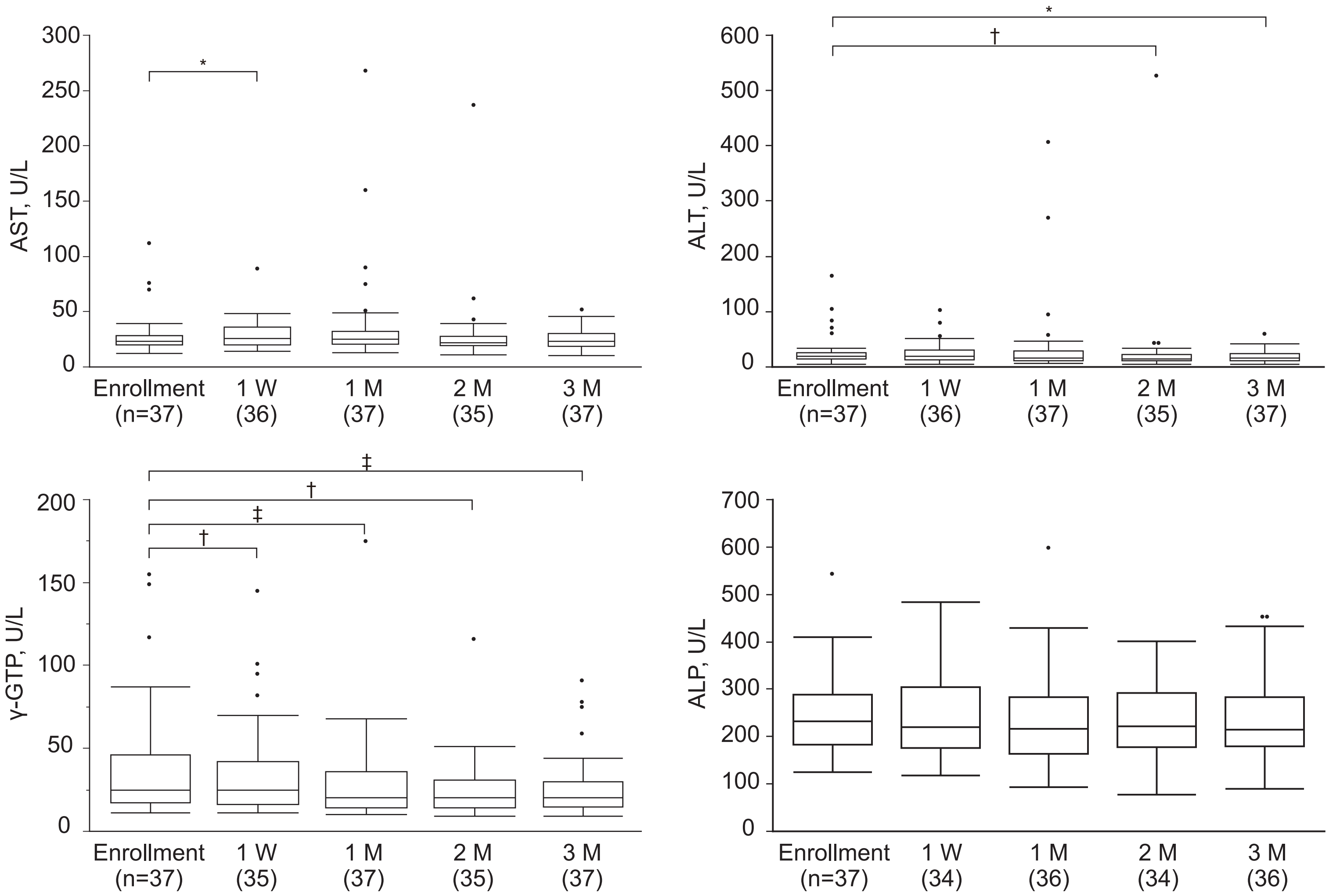

*P < 0.05, †P < 0.01, ‡P < 0.001

Figure S2-4 : Additional blood parameters of patients on the ketogenic diet.

Abbreviations; ALP, Alkaline Phosphatase; ALT, Alanine Aminotransferase; AST, Aspartate aminotransferase; BUN, Blood Urea Nitrogen; Cl, Chloride; Cre, Creatinine; eGFR, Estimated Glomerular Filtration Rate; Hb, Hemoglobin; HbA1c, Hemoglobin A1c;HDL, High Density Lipoprotein Cholesterol; Ht, Hematocrit; K, Potassium; LDL, Low Density Lipoprotein Cholesterol; Na, Sodium;Plt, Platelet; RBC, Red blood cell/coun; T-Cho, Total Cholesterol; TG, Triglycerides; UA, Uric acid; WBC, While blood count/cell; γ-GTP, Gamma-glutamyl transpeptidase; 1,5-AG, 1,5 Anhydroglucitol;

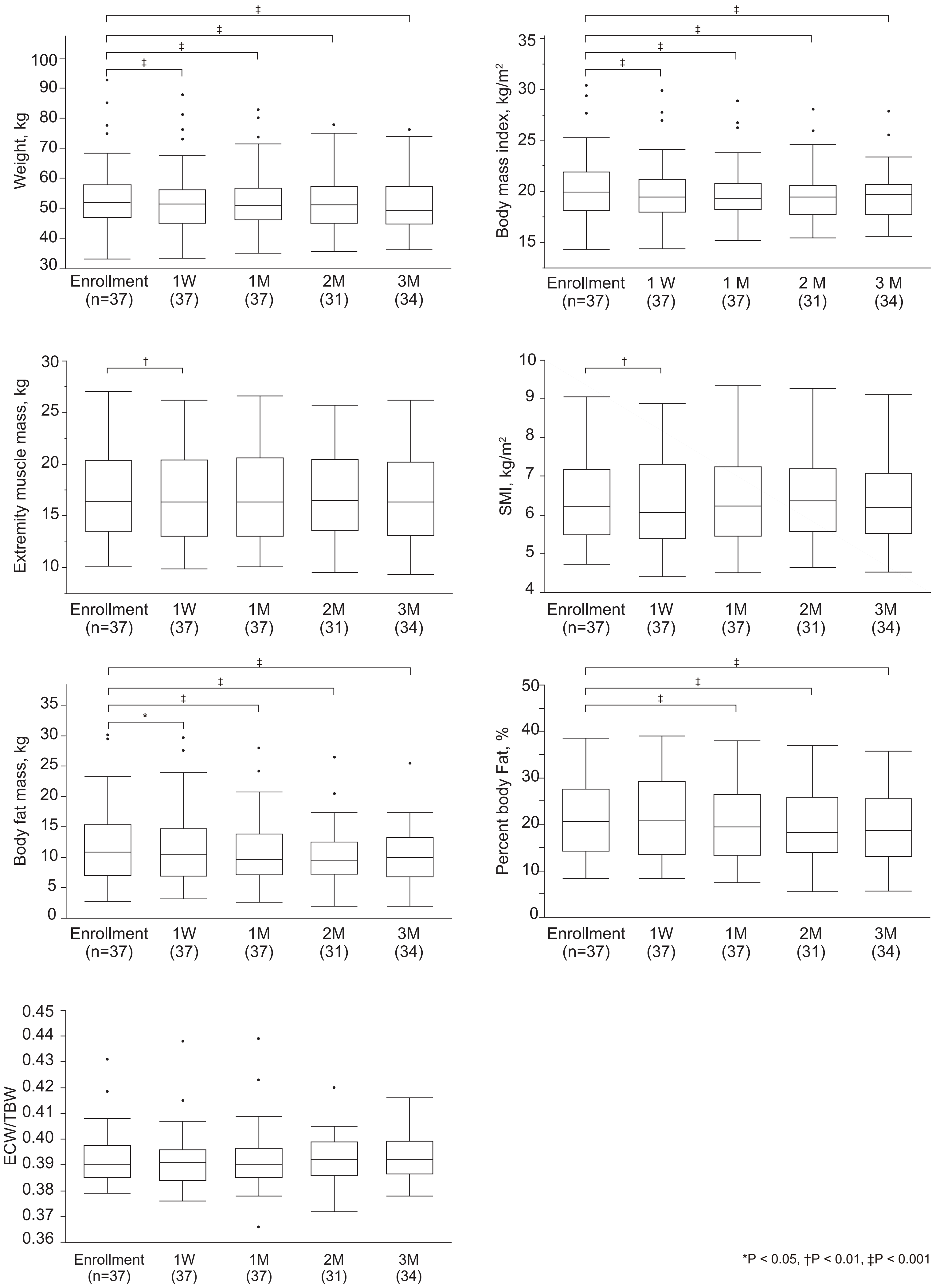

Figure S3 : Changes of body composition evaluated with the In body 720 on the ketogenic diet.

Table S1: Nutritional contents of the ketogenic formula (per 100 g).

	Ketogenic formula
Carbohydrates (g)	8.8
Carbohydrates (% kcal)	4.7
Proteins (g)	15.0
Protein (% kcal)	8.1
Lipids (g)	71.8
MCT (g)	39.7
Lipids (% kcal)	87.2
Energy (kcal)	741
Vitamins	
Vitamin A (μg RE)	600
Vitamin B1 (mg)	0.6
Vitamin B2 (mg)	0.9
Vitamin B6 (mg)	0.3
Vitamin B12 (μg)	4
Vitamin C (mg)	50
Vitamin D (μg)	12.5
Vitamin E (mg α-TE)	6
Vitamin K (μg)	30
Niacin (mg)	6
Folic acid (μg)	200
**Minerals**	
Sodium (mg)	160
Potassium (mg)	470
Calcium (mg)	350
Magnesium (mg)	36
Phosphorus (mg)	240
Iron (mg)	6
Zinc (mg)	2.6
Copper (mg)	0.35
Chloride (mg)	320

MCT, medium chain triglycerides; RE, retinol equivalent; TE, tocopherol equivalent.

Table S2 : Background of patients (n = 55).

Age, y	55.8 ± 12.1
Sex (male/ female), n	24/ 31
Body height, cm	162.3 ± 8.7
Body weight, kg	54.7 ± 12.1
Body mass index, kg/m^2	20.6 ± 3.3
**Primary cancer**	
Non-small cell lung cancer, n	15
Colorectal cancer, n	9
Breast cancer, n	6
Pancreatic cancer, n	5
Head and neck cancer, n	4
Bone and soft tissue sarcoma, n	4
Ovarian cancer and peritoneal cancer, n	3
Bladder cancer, n	2
Biliary tract cancer, n	2
Endometrial cancer, n	1
Brain tumor, n	1
Gastric cancer, n	1
Prostate cancer, n	1
Esophageal cancer, n	1
**Treatment history**	
Chemohormonal therapy, n (%)	50 (90.9)
Radiation therapy, n (%)	20 (36.4)
Surgical therapy, n (%)	37 (67.3)

Data are presented as mean ± SD or number for continuous and categorical variables, respectively.

Table S3-1 : Histological and therapeutic background of patients (n = 37).

Paitient No	Sex	Age	Cancer type	Histology	TNM classification	Chemohormonal therapy	Surgical therapy	Radiation therapy
1	F	56	Lung cancer	Adenocarcinoma	T2aN0M1a	CBDCA + PEM + BEV(6 courses), PEM + BEV(4 courses)	N/A	N/A
2	M	65	Lung cancer	Adenocarcinoma	T2aN0M1a	CDDP + PEM, PEM(20 courses), **DTX**	Left upper pulmonary lobectomy	N/A
3	F	52	Lung cancer	Adenocarcinoma	T2aN0M1b	UFT, **ERL**	Left lower pulmonary lobectomy, Resection for left metastatic temporal lobe brain tumor	Gamma knife for brain metastasis
4	F	69	Breast cancer	Invasive ductal carcinoma	T4N3bM1	UFT + TAM, TAM + 5'-DFUR, TOR + 5'-DFUR, TOR, ANA, LET, EXE, MPA, FUL, TAM, **Capecitabine, Capecitabine +CPA**	Mastectomy + ALND, Chest wall resection	50Gy/25fr, 40Gy/20fr, 60Gy/25fr
5	F	65	Endometrial cancer	Clear cell adenocarcinoma	T3aN0M1	PTX + DXR + CBDCA(6 courses), GEM + 5-FU + l-LV + CPT-11(3 courses)	TAH + BSO + PALA + PLA + omentectomy + peritonectomy	N/A
6	F	50	Peritoneal cancer	Serous adenocarcinoma	T3aN1M1	PTX + CBDCA(18 courses), DTX + CBDCA(7 courses), **PTX + BEV**	Anterior resection of rectum + left salpingo-oophorectomy + right salpingectomy, TAH + right oophorectomy + PALA + PLA + omentectomy	N/A
7	F	29	Colorectal cancer	Tubular adenocarcinoma	T4bN2bM1a	**mFOLFOX6**	**Rectectomy**	N/A
8	M	71	Bladder cancer	Urothelial carcinoma	T3bN3M1	GEM + NDP(2 courses), **GEM + CDDP**(2 courses and more)	Total cystectomy + radical prostatectomy + ileal conduit urinary diversion, Partial nephrectomy, Bipolar hip arthroplasty for bone , Metastasis	45Gy/15fr for bone metastasis
9	M	46	Oral and pharyngeal cancer	Adenoid cystic carcinoma	T4aN1M1	DTX + CDDP + Cmab(2 course) **DTX + Cmab + Denosumab**	Resection for lip cancer	60Gy/30fr
10	M	71	Colorectal cancer	Mucinous adenocarcinoma	T4aN2aM1b	Capecitabine, S-1, **SOX**(4 corses) **BEV + FOLFOX6**	Laparoscopic right hemicolectomy + super-extended (D3), Lymphadenectomy,Resection for peritoneal metastasis	N/A
11	F	36	Colorectal cancer	Tubular adenocarcinoma	T4bN1aM1b	S-1 + PSK, FOLFOX(12 courses), **Capecitabine + CPT-11**(3 courses)	Laparoscopic anterior resection for colorectal cancer, Laparoscopic segmentectomy for liver metastasis, Low anterior resection for anastomotic site recurrence, BSO + pelvic peritonectomy for ovarian metastasis, TAH + pelvic peritonectomy + appendectomy + omentectomy for metastatic lesions	N/A
12	F	65	Colorectal cancer	Tubular adenocarcinoma	T3N2bM1b	PTX + Trastuzumab + Pertuzumab, FOLFOX + BEV, **FOLFIRI + BEV**	Laparoscopic low anterior, Resection for rectal cancer, Segmentectomy for liver metastasis,Limited resection for lung metastasis	N/A
13	F	36	Oral and pharyngeal cancer	Adenoid cystic carcinoma	T4bN0M1	N/A	N/A	70.4Gy/32fr
14	F	41	Breast cancer	Invasive ductal carcinoma	T3N1M1	**TAM + ZOL**	N/A	N/A
15	M	79	Sarcoma	Chondrosarcoma	T2N1M1	N/A	Excision for chondrosarcoma+ total hip arthroplasty, Wide excision for local recurrence + total hip arthroplasty, Excision for pubic bone metastasis, Thoracoscopic left lower pulmonary lobectomy for lung metastasis	N/A
16	M	46	Sarcoma	Chondrosarcoma	T2N0M1	**Pazopanib**	Wide excision for chondrosarcoma Radiofrequency ablation	N/A
17	F	49	Breast cancer	Invasive ductal carcinoma	T2N1M1	PTX(12 courses), FEC(4 courses), S-1, GnRH agonist, TAM, ANA + GnRH agonist, **PTX + BEV**	Quadrantectomy for breast cancer+ALND	50Gy/25fr
18	F	63	Lung cancer	Adenocarcinoma	T1cN2M1c	**Afatinib**	Resection for brain metastasis	N/A

Table S3-2 : Histological and therapeutic background of patients (n = 37).

Paitient No	Sex	Age	Cancer type	Histology	TNM classification	Chemohormonal therapy	Surgical therapy	Radiation therapy
19	F	50	Ovarian cancer	Mucinous adenocarcinoma	T3bN0M1	PTX + CBDCA(8 courses)<br>**Denosumab**	Laparoscopic left salpingo-oophorectomy, TAH + right salpingo-oophorectomy + PALA + PLA + omentectomy	N/A
20	M	44	Brain tumor	Anaplastic astroblastoma	-	N/A	Resection for brain tumor	**+(details unknown)**
21	M	55	Oral and pharyngeal cancer	Squamous cell carcinoma	T1N2M1	DTX + CDDP(6 courses), DTX + CDDP + Cmab(6 courses), Cmab + DTX + CDDP, Cmab, **Nivolumab**	N/A	70Gy/35fr
22	F	48	Sarcoma	Leiomyosarcoma	T1NXM1	GEM + DTX(4 courses), DXR(4 courses), Pazopanib, **Eribulin**	THA	N/A
23	F	76	Pancreatic cancer	Adenocarcinoma	T3N0M1	**GEM + nab-PTX**	N/A	N/A
24	F	74	Pancreatic cancer	Adenocarcinoma	T2N0M1	GEM(11 courses), **S-1**	N/A	N/A
25	M	55	Lung cancer	Adenocarcinoma	T3N1M1a	**Crizotinib**	N/A	N/A
26	M	39	Colorectal cancer	Tubular adenocarcinoma	T3N2aM1a	N/A	Endoscopic submucosal dissection for rectal cancer	N/A
27	F	46	Colorectal cancer	Tubular adenocarcinoma	T4aN0M1c	Capecitabine + L-OHP(6 courses), Capecitabine(12 courses), IRIS + BEV(17 courses), mFOLFOX6(16 courses), **TAS-102**	Sigmoidectomy + TAH + BSO + omentectomy + appendectomy, Thoracic spinal fixation for spinal metastasis	50Gy/10fr for bone metastasis<br>36.3Gy/11fr for bone metastasis<br>30-40Gy/10fr for bone metastasis<br>40Gy/10fr for bone metastasis<br>40Gy/10fr for bone metastasis
28	F	62	Pancreatic cancer	Adenocarcinoma	T4N0M1	FOLFIRINOX(4 courses), **GEM + nab-PTX**	Irreversible electroporation	N/A
29	M	80	Cholangiocarcinoma	Adenocarcinoma	T4N1M1	GEM + CDDP, GEM, **S-1**	Endoscopic placement of self-expandable metal stents	N/A
30	F	50	Colorectal cancer	Tubular adenocarcinoma	TXN1bM1a	Capecitabine + L-OHP(6 courses), Capecitabine, **FOLFIRI**	Laparoscopic sigmoidectomy	N/A
31	M	47	Gastric cancer	Adenocarcinoma	T4aN3M1	S-1 + DTX + L-OHP(8 courses),<br>S-1 + DTX(2 courses), **nab-PTX + Ramucirumab**	N/A	N/A
32	M	54	Prostate cancer	Adenocarcinoma	T3aN1M1a	Degarelix, GnRH antagonist + Bicalutamide,<br>**GnRH agonist, Abiraterone**	N/A	45Gy/25fr
33	F	54	Breast cancer	Invasive ductal carcinoma	T2N0M1	ZOL, TAM, **ZOL + FUL + Palbociclib, Denosumab**	Quadrantectomy for left breast cancer,<br>Quadrantectomy for right breast cancer	50Gy/25fr<br>50Gy/25fr
34	M	58	Colorectal cancer	Tubular adenocarcinoma	T3N0M1	**mFOLFOX6, mFOLFOX6 + Pmab**	Sigmoidectomy	N/A
35	F	50	Lung cancer	Adenocarcinoma	T4N1M1c	Alectinib, **Ceritinib**	Pleural fluid drainage	Gamma knife for brain metastasis
36	M	48	Pancreatic cancer	Adenocarcinoma	T3N0M1	**GEM + nab-PTX, S-1**	N/A	N/A
37	F	49	Breast cancer	Invasive ductal carcinoma	T2N0M1	PTX + FEC, TAM, LET, FUL,<br>Palbociclib + LET, Everolimus + EXE, **Capecitabine, Denosumab**	Lumpectomy for right breast cancer	50Gy/25fr<br>30Gy/10fr for bone metastasis<br>30Gy/10fr for bone metastasis

Abbreviations for Chemotherapy; ANA, Anastrozol; BEV, Bevacizumab; CBDCA, Carboplatin; CDDP, Cisplatin; Cmab, Cetuximab; CPA,cyclophosphamide; CPT-11, Irinotecan hydrochloride hydrate; DTX, Docetaxel hydrate; DXR, doxorubicin; EPI, Epirubicin hydrochloride; ERL, Erlotinib; EXE, Exemestane; FEC, 5-FU+EPI+CPA; FOLFIRI, 5-FU+l-LV+CPT-11, FOLFIRINOX, 5-FU+l-LV+CPT-11+L-OHP; FOLFOX, 5-FU+l-LV+L-OHP; FUL, Fulvestrant; GEM, Gemcitabine hydrochloride; GnRH, Gonadotropin-releasing hormone; IRIS, IRI(CPT-11)+S-1; LET, Letrozole; l-LV, Levofolinate calcium; L-OHP, Oxaliplatin; mFOLFOX6, 5-FU+l-LV+L-OHP; MPA, Medroxyprogesterone acetate; nab-PTX, Nab-Paclitaxel; NDP, Nedaplatin; PEM, Pemetrexed sodium hydrate; Pmab,Panitumumab; PSK, Polysaccharide-Kureha; PTX, Paclitaxel; S-1, Tegafur/Gimeracil/Oteracil potassium; SOX, S-1+L-OHP; TAM, Tamoxifen citrate; TAS-102, Trifluridine + Tipiracil hydrochloride; TOR, Toremifene citrate; UFT, Tegfur/Uracil; ZOL, Goserelin acetate; 5'-DFUR, Doxifluridine; 5-FU, 5-fluorouracil

Abbreviations for Surgical therapy; ALND, axillary lymph node dissection; BSO, bilateral salpingo-oophorectomy; PLA: pelvic lymphadenectomy;PALA, para-aortic lymphadenectomy; TAH, total abdominal hysterectomy

Bold text indicates other treatments performed during ketogenic diet

Table S4 : Glucose, BHB, and GKI data of 37 patients after ketogenic diet.

Paitient No	Glucose(mg/dL)					BHB(μmol/L)					GKI				
	Enrollment (n=37)	1W (n=37)	1M (n=37)	2M (n=35)	3M (n=37)	Enrollment (n=37)	1W (n=37)	1M (n=37)	2M (n=36)	3M (n=37)	Enrollment (n=37)	1W (n=37)	1M (n=37)	2M (n=35)	3M (n=37)
1	77	107	96		105	17	2169	1486	1851	597	251.4	2.7	3.6		9.8
2	96	82	80	81	79	1381	2906	3762	2564	2757	3.9	1.6	1.2	1.8	1.6
3	97	71	84	75	79	147	4010	1646	2833	2870	36.6	1.0	2.8	1.5	1.5
4	98	94	99	105	100	125	2318	538	942	672	43.5	2.3	10.2	6.2	8.3
5	110	116	104	106	111	1827	3043	4038	3284	6385	3.3	2.1	1.4	1.8	1.0
6	101	123	108	99	98	56	2101	1167	1031	538	100.1	3.2	5.1	5.3	10.1
7	91	83	77		95	266	1162	982		65	19.0	4.0	4.4		81.1
8	107	114	107	119	108	28	3491	2730	1916	1204	212.1	1.8	2.2	3.4	5.0
9	84	77	96	94	88	89	2107	1027	1129	1269	52.4	2.0	5.2	4.6	3.8
10	111	110	92	106	107	114	2107	1265	611	896	54.0	2.9	4.0	9.6	6.6
11	89	76	74	60	82	22	2588	1511	3988	794	224.5	1.6	2.7	0.8	5.7
12	101	80	99	88	91	148	3527	444	1223	193	37.9	1.3	12.4	4.0	26.2
13	81	68	69	68	58	397	2777	2297	1508	2681	11.3	1.4	1.7	2.5	1.2
14	92	78	94	85	78	421	3016	488	1038	2416	12.1	1.4	10.7	4.5	1.8
15	100	60	74	79	73	19	5103	3772	3366	4222	292.1	0.7	1.1	1.3	1.0
16	88	80	81	77	91	129	1868	2195	2014	498	37.9	2.4	2.0	2.1	10.1
17	100	69	76	81	75	57	2881	1924	1567	1955	97.4	1.3	2.2	2.9	2.1
18	103	87	88	89	86	220	1034	2431	1300	1235	26.0	4.7	2.0	3.8	3.9
19	95	85	85	93	107	131	1650	1679	2232	3055	40.3	2.9	2.8	2.3	1.9
20	91	64	88	70	80	1243	4757	470	2278	1472	4.1	0.7	10.4	1.7	3.0
21	100	83	98	95	99	172	1441	506	582	379	32.3	3.2	10.8	9.1	14.5
22	90	79	92	92	81	39	1651	895	909	1319	128.1	2.7	5.7	5.6	3.4
23	104	92	89	79	95	229	1685	1510	2673	1249	25.2	3.0	3.3	1.6	4.2
24	121	119	111	122	119	308	653	1380	987	1408	21.8	10.1	4.5	6.9	4.7
25	103	86	82	90	90	41	1963	3633	3107	2242	139.4	2.4	1.3	1.6	2.2
26	95	118	115	98	92	1149	1228	1081	863	1640	4.6	5.3	5.9	6.3	3.1
27	82	74	77	78	82	327	1943	1219	476	1431	13.9	2.1	3.5	9.1	3.2
28	85	92	86	88	87	19	2403	2671	2765	3087	248.3	2.1	1.8	1.8	1.6
29	108	93	95	98	94	116	1739	735	1139	1315	51.7	3.0	7.2	4.8	4.0
30	88	87	76	90	89	606	1746	3294	1854	2245	8.1	2.8	1.3	2.7	2.2
31	97	80	96	113	107	211	4061	1391	691	433	25.5	1.1	3.8	9.1	13.7
32	97	84	79	83	74	204	3838	3709	2992	3770	26.4	1.2	1.2	1.5	1.1
33	96	88	81	85	88	56	1801	2026	1821	1208	95.2	2.7	2.2	2.6	4.0
34	105	92	95	97	99	445	3774	691	1224	418	13.1	1.4	7.6	4.4	13.1
35	82	73	65	84	89	951	5067	3929	1310	1016	4.8	0.8	0.9	3.6	4.9
36	97	88	90	89	112	417	2496	1152	1293	949	12.9	2.0	4.3	3.8	6.6
37	98	87	95	105	94	52	3526	3645	739	964	104.6	1.4	1.4	7.9	5.4

Table S5 : Gastrointestinal symptoms rating scale (GSRS).

		Elapsed time after enrollment (months)		
	Enrollment (n = 37)	1 (n = 36)	2 (n = 35)	3 (n = 34)
GSRS total	1.64 ± 0.53	1.95 ± 0.70†	1.92 ± 0.78	1.80 ± 0.76
Reflux	1.23 ± 0.40	1.58 ± 0.89*	1.50 ± 0.93	1.60 ± 0.90*
Abdominal pain	1.45 ± 0.67	1.65 ± 0.81	1.73 ± 1.17	1.67 ± 0.95
Indigestion	1.58 ± 0.68	1.63 ± 0.73	1.56 ± 0.80	1.56 ± 0.84
Diarrhea	1.55 ± 0.86	1.73 ± 1.25	1.78 ± 1.17	1.74 ± 0.97
Constipation	2.40 ± 1.31	3.15 ± 1.27*	3.03 ± 1.54*	2.45 ± 1.22

Paired *t*-tests; Enrollment vs. 1, 2 and 3 months. *$P<0.05$, †$P<0.01$

Table S6 : EORTC-QLQ-c30

	Elapsed time after enrollment (months)			
	Enrollment (n = 37)	1 (n = 36)	2 (n = 35)	3 (n = 34)
Global health	59.7 ± 24.7	60.5 ± 26.3	61.8 ± 24.7	56.5 ± 26.9
Financial difficulties	31.2 ± 33.3	21.5 ± 30.5*	25.8 ± 29.5	23.7 ± 24.6
Physical functioning	86.0 ± 15.6	81.3 ± 18.1†	80.0 ± 16.3*	79.4 ± 20.7*
Role functioning	80.7 ± 30.2	75.3 ± 29.8	76.3 ± 27.5	72.6 ± 30.3
Emotional functioning	77.4 ± 20.0	79.0 ± 20.1	80.7 ± 17.7	80.9 ± 19.9
Cognitive functioning	81.2 ± 23.5	79.0 ± 24.3	78.0 ± 22.9	79.0 ± 23.6
Social functioning	69.4 ± 30.2	71.5 ± 31.1	70.4 ± 30.0	71.0 ± 27.5
Fatigue	34.4 ± 20.0	37.6 ± 21.6	35.5 ± 26.1	40.9 ± 26.2
Pain	14.5 ± 19.1	18.3 ± 23.7	18.8 ± 25.4	22.0 ± 29.0
Dyspnea	11.8 ± 16.2	18.3 ± 20.8	17.2 ± 19.0	20.4 ± 20.5*
Insomnia	29.0 ± 35.2	24.7 ± 28.5	26.9 ± 30.3	23.7 ± 30.1
Nausea and vomiting	5.4 ± 10.0	9.7 ± 15.4	9.7 ± 22.3	5.9 ± 12.6
Appetite loss	22.6 ± 27.7	19.4 ± 28.3	21.5 ± 36.1	29.0 ± 33.1
Constipation	20.4 ± 28.1	39.8 ± 32.7†	34.4 ± 30.4*	25.8 ± 25.4
Diarrhea	9.7 ± 26.1	12.9 ± 23.9	11.8 ± 26.6	11.8 ± 23.7

Data are presented as means ± SD. EORTC indicates European Organization for Research and Treatment of Cancer; QLQ, quality of life questionnaire module. Paired *t*-tests; Enrollment vs. 1, 2 and 3 months, *$P<0.05$, †$P<0.01$
